# Development of a Paper-Based Microfluidic Chip for Point-of-Care Detection of PEDV

**DOI:** 10.3390/vetsci12050427

**Published:** 2025-04-30

**Authors:** Renfeng Li, Xiangqin Tian, Wenyan Cao, Jiaxin Jiang, Jiakang Yuan, Linyue Li, Yonghe You, Yanlin Zhou, Ziliang Wang, Fangyu Wang

**Affiliations:** 1 College of Animal Science and Veterinary Medicine, Henan Institute of Science and Technology, Xinxiang 453003, China; lirenfeng@sina.com (R.L.); caowenyan@stu.hist.edu.cn (W.C.); jiangjiaxin@stu.hist.edu.cn (J.J.); yjkhist2024@163.com (J.Y.); m18236356245@163.com (L.L.); 2 Henan Key Laboratory of Medical Tissue Regeneration, Xinxiang Medical University, Xinxiang 453003, China; tianxiangqin@xxmu.edu.cn; 3 Sanquan College, Xinxiang Medical University, Xinxiang 453003, China; sqjcyyh2013@126.com (Y.Y.); zhouyanlin@xxmu.edu.cn (Y.Z.); 4 Key Laboratory of Animal Immunology, Institute for Animal Health, Henan Academy of Agricultural Sciences, Zhengzhou 450002, China

**Keywords:** porcine epidemic diarrhea virus, loop-mediated isothermal amplification, paper-based microfluidic analytical device, point-of-care detection

## Abstract

PEDV poses a significant threat to the global swine industry, necessitating rapid and accurate diagnostic methods for effective disease management. In this study, we developed a foldable, easy-to-use paper-based microfluidic analytical device (*μ*PAD) for on-site detection of PEDV. The device seamlessly integrates paper-based nucleic acid enrichment, LAMP reaction, and visual lateral flow detection into a single platform. Key parameters, including nucleic acid extraction protocols, chromatographic channel configurations, colorimetric indicators, and reaction temperature and duration, were systematically optimized. The resulting LAMP-*μ*PAD assay detects PEDV within 30 min at 60 °C, achieving a limit of detection of 4.82 × 10^2^ copies/μL with no cross-reactivity against other viruses. When evaluated against RT-PCR using clinical specimens, the assay demonstrated a specificity of 100%, a sensitivity of 95.3%, and an overall concordance of 98.5%. This paper-based sensor offers a promising alternative for the rapid, on-site detection of PEDV and other highly transmissible pathogens.

## Simple Summary

This study introduces an innovative cut-and-stack 3D microfluidic device for point-of-care detection of the porcine epidemic diarrhea virus (PEDV), utilizing a foldable paper chip coupled with loop-mediated isothermal amplification (LAMP) and a pH-responsive indicator. By integrating viral nucleic acid enrichment, amplification, and visual quantitative detection within a single device, this assay effectively addresses the limitations of conventional PEDV diagnostic techniques for field applications. Furthermore, it demonstrates significant potential for broader application in the detection of other pathogens, particularly in resource-limited settings.

## 1. Introduction

PEDV causes an acute, highly contagious enteric disease in swine, characterized by vomiting, watery diarrhea, and dehydration [1]. While PEDV infects swine of all age groups, it exhibits particularly severe mortality rates approaching 100% in neonatal piglets less than seven days old [2]. Since its first identification in the UK in 1971, PEDV has spread globally, causing significant economic losses to the pork industry [3,4]. The rapid transmission and devastating impact of PEDV underscore the critical need for effective on-site diagnostic tools.

Currently employed methods for PEDV detection primarily relies on virus isolation, nucleic acid amplification, and serological techniques [5,6,7]. Virus isolation, though reliable, is time-consuming and impractical for field use. Nucleic acid detection methods, including reverse transcription-polymerase chain reaction (RT-PCR) and real-time quantitative RT-PCR (qPCR), offer high sensitivity but require specialized equipment and a large amount of reagents and expertise. Serological tests, such as neutralization assays and enzyme-linked immunosorbent assay (ELISA), generally exhibit lower specificity and sensitivity compared to nucleic acid detection methods. These limitations highlight the pressing necessity to develop rapid, straightforward, and user-friendly diagnostic tools that are appropriate for field implementation, thereby improving the detection and management of PEDV.

Recent advances in nucleic acid isothermal amplification techniques have led to the emergence of LAMP as an innovative alternative to traditional thermal cycling methods. Utilizing Bst DNA polymerase and 4–6 specially designed primers, LAMP enables rapid target sequence amplification under constant temperature conditions [8,9]. and the results can be visualized through turbidity, dye-based methods, or immunochromatographic strips [10,11,12]. LAMP demonstrates high specificity due to its targeting of six distinct regions and offers rapid amplification using simple, cost-effective equipment, making it a promising tool for on-site diagnostics in resource-limited settings [13].

Microfluidic chips represent an innovative diagnostic platform that enables rapid sample analysis through the utilization of microliter-scale reaction volumes. *μ*PADs constitute an advanced evolution of conventional microfluidic chips, utilizing paper as the foundational substrate material. These devices integrate precisely engineered microchannels fabricated on diverse paper types, leveraging capillary action to autonomously drive fluid flow without external energy input [14,15]. This approach offers a more cost-effective and simplified solution for analytical purposes. To date, *μ*PADs have demonstrated efficacy in the detection of diverse human and animal pathogens, including porcine reproductive and respiratory syndrome virus (PRRSV) [16], pseudorabies virus (PRV) [17], human papillomavirus [18], severe acute respiratory syndrome coronavirus 2 (SARS-CoV-2) [19,20], Zika virus (ZIKV) [21], Avian influenza virus (AIV) [22], and mycobacterium tuberculosis [23], among others. In this study, we introduce the integration of LAMP with paper-based microfluidic technology to develop an innovative LAMP-*μ*PAD assay for the detection of PEDV. This approach demonstrates high accuracy, specificity, and sensitivity, while maintaining cost-effectiveness and ease of use.

## 2. Materials and Methods

### 2.1. Materials, Reagents and Instruments

PEDV, porcine deltacoronavirus (PDCoV), porcine circovirus (PCV2), PRRSV, porcine transmissible gastroenteritis virus (TGEV), and standard plasmid harboring the PEDV ORF1a/b gene were obtained from the Key Laboratory of Animal Viral Diseases Prevention and Control at Henan Institute of Science and Technology. WarmStart^®^ RTx reverse transcriptase and Bst 2.0 WarmStart^®^ DNA polymerase were purchased from NEB (Beijing, China). Methyl red and neutral red indicator dyes were purchased from Macklin Co., Ltd. (Shanghai, China). Whatman No. 1 filter paper and GF/F glass fiber membrane were purchased from GE Healthcare Life Sciences (Pittsburgh, PA, USA). Nitrocellulose (NC) membrane (HiFlow Plus 180) was purchased from Merck Millipore Ltd. (Burlington, VT, USA); The absorbent paper pad (Grade 222) was purchased from Ahlstrom (Helsinki, Finland); Portrait 3 electronic cutting machine was purchased from Silhouette (Lindon, UT, USA).

### 2.2. Optimization of RT-LAMP Reaction System

The reaction mixture comprised 2.5 μL of 10× isothermal amplification buffer, 1.5 μL of MgSO_4_ (100 mM), 3.5 μL of dNTPs Mix (10 mM), 1 μL of Bst 2.0 WarmStart DNA polymerase (8000 U/mL), 1 μL each of F3/B3 primers (5 μM), 1 μL each of FIP/BIP primers (40 μM), 1 μL of standard plasmid as the template, and nuclease-free water to a final volume of 25 μL. Utilizing the LAMP reaction system, a comprehensive screening of 11 primer sets was conducted to select the most suitable candidates for subsequent visual RT-LAMP assay (Table S1). Concurrently, we undertook an optimization process for various parameters implicated in the LAMP reaction, including reaction time, reaction temperature, concentrations of inner and outer primers, Bst 2.0 enzyme concentration, Mg^2+^ concentration, dNTPs concentration, and buffer concentration. The optimal conditions were determined based on agarose gel electrophoresis results.

### 2.3. Fabrication of the μPAD

**Figure 1:**
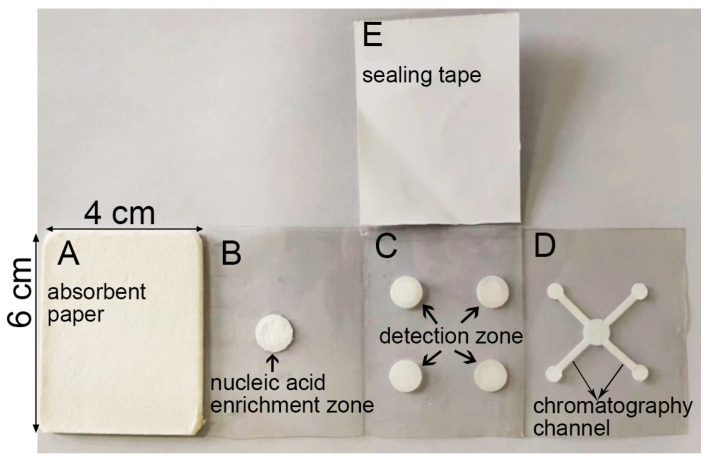
Photograph of the LAMP-*μ*PAD. (**A**) Waste liquid zone. (**B**) Nucleic acid enrichment zone. (**C**) Nucleic acid amplification zone. (**D**) Nucleic acid chromatography zone. (**E**) Film sealing tape.

The paper chip architecture was designed using AutoCAD 2016 software (Autodesk, San Francisco, CA, USA), incorporating multiple functional zones. The design was exported to Silhouette Studio software V4.4.259.001 for fabrication using a Portrait 3 cutting machine. As illustrated in Figure 1, the paper-based chip comprises five 4 × 6 cm functional modules. Module A is a waste reservoir made of absorbent paper that collects impurities eluted from the enrichment zone. Module B contains a glass fiber membrane disk for enriching viral nucleic acids. Module C includes four filter paper detection zones for performing LAMP amplification and subsequent colorimetric detection. Module D features a nucleic acid chromatography channel for transferring nucleic acids from the enrichment zone to the amplification zone. Module E is a sealing tape that prevents evaporation and cross-contamination during the LAMP amplification reaction. All components were precision-cut and assembled using adhesive tape, resulting in an integrated design that enables efficient nucleic acid enrichment, amplification, and detection within a single paper-based device.

### 2.4. Selection of Paper Substrate

Circular discs of Whatman No. 1 filter paper and NC membrane were precision-cut using a Portrait 3 cutter, and oriented horizontally in 1.5 mL microcentrifuge tubes, respectively. LAMP reaction mixture and DNA template (PEDV cDNA) were dispensed onto each disc, followed by incubation at 60 °C for 40 min. The amplification products were separated from the paper matrix by centrifugation at 13,000× *g* for 1 min. After removing the paper, concentrated products were analyzed by 1% agarose gel electrophoresis. Based on electrophoretic results, the substrate showing optimal amplification efficiency was selected as the base material for the nucleic acid amplification zone in the paper-based chip design.

### 2.5. Optimization of Viral Nucleic Acid Lysis Buffer

The extraction and release of viral nucleic acids play a crucial role in establishing paper-based chip detection methods. To enhance the efficiency of this process, an optimization of the virus lysis buffer was conducted prior to LAMP reaction. The PEDV propagated in Vero cell culture was equally divided into three aliquots, each subjected to lysis using one of three distinct lysis buffers: buffer I (4.5 M guanidine thiocyanate, 50 mM Tris-HCl, 30% Triton X-100, and 1 mg/mL proteinase K), buffer II (6 M guanidine hydrochloride, 1% Tween, and 1% SDS), and buffer III (3 M guanidine hydrochloride and 4 M urea). Subsequently, nucleic acid enrichment was performed using glass fiber membranes.

To evaluate the efficacy of each lysis buffer, the extracted nucleic acids underwent a series of analytical procedures, including RT-PCR, qPCR, and RNA agarose gel electrophoresis. The results obtained from these assays were comprehensively analyzed to determine the optimal virus lysis buffer for the paper-based chip detection system.

### 2.6. Optimization of the Chromatography Channel and Nucleic Acid Amplification Zone Geometry

The optimization of the nucleic acid chromatography channels on the paper chip involved adjusting both their lengths and widths. Initially, various channel lengths (12, 16, 20, 24, 28, and 32 mm) were tested by adding 30 μL of blue ink. The time taken for the ink to fill the channel and the residual ink in the sample well were observed and recorded to determine the most suitable length. Using this optimal length, different channel widths (2, 3, 4, 5, and 6 mm) were then tested with 30 μL of blue ink. The time required for the ink to fill the channel and the residual ink in the well were again observed and recorded to identify the optimal width.

To determine the optimal size of the reaction well, a series of experiments were conducted using wells with varying diameters ranging from 7 mm to 18 mm. In each trial, 30 μL of blue ink was deposited onto the reaction well. The extent of coverage and the amount of residual ink were recorded and analyzed. This systematic approach allowed for the identification of the most suitable reaction well size, balancing complete reagent distribution with minimal waste.

### 2.7. Optimization of Indicator

The selection of an appropriate pH-sensitive indicator is crucial for the visual detection of nucleic acid amplification in paper-based microfluidic chips. Two candidate dyes were evaluated: cresol red, which exhibits a color transition from purple–red to yellow over a pH range of 7.2 to 8.8, and neutral red, which changes from yellow–orange to magenta within a pH range of 6.4 to 8.0. To determine the most suitable indicator, both cresol red and neutral red were independently employed in LAMP amplification reactions, conducted both in solution and on paper-based microfluidic chips. The efficacy of each dye was assessed based on two criteria: (1) the distinctiveness of the color change, and (2) the correlation between the visual results and the amplification products as verified by 1% agarose gel electrophoresis. This comprehensive evaluation enabled the identification of the optimal colorimetric indicator for the LAMP-*μ*PAD assay.

### 2.8. Detection Procedure of the LAMP-μPAD

The operational procedure is delineated as follows: (1) Clinical fecal specimens are deposited into 1.5 mL centrifuge tubes, to which 500 μL of 0.9% NaCl (*w*/*v*) is added. The mixture is centrifuged at 5180× *g* for 10 min, after which the supernatant is transferred to a new collection tube. (2) A 200 μL aliquot of the supernatant is transferred to a 1.5 mL centrifuge tube, followed by the addition of 200 μL of lysis buffer. This mixture is incubated at room temperature for 10 min. (3) Module A of the paper-based device is folded beneath Module B. The lysis mixture is then slowly applied to the glass fiber membrane in Module B (nucleic acid enrichment zone) and allowed to incubate for 2–3 min. (4) 200 μL of washing solution A (20 mM Tris-HCl, 40% (*v*/*v*) ethanol, pH 6.6) is added onto nucleic acid enrichment zone in Module B to remove extraneous proteins, with Module A absorbing the waste liquid. (5) 200 μL of washing solution B (50% (*v*/*v*) ethanol, 20 mM NaCl, 2 mM Tris-HCl, pH 7.5) is applied to eliminate high salt concentrations, with Module A again absorbing the waste liquid. (6) Step (5) is repeated, followed by the excision of Module A. (7) RT-LAMP reaction mixture, including reverse transcriptase, Bst polymerase, primers, dNTPs, Mg^2+^ and indicator, are pre-loaded to detection zone in Module C. Following the indicated direction, Module D is folded onto Module C, and Module B is then folded onto Module D. A total of 200 μL of isothermal amplification buffer is applied centrally to elute nucleic acids. (8) Once the reagents have fully permeated the detection zone in Module C, Module B and D are excised, and Module E is folded downward to seal the device. (9) The paper-based microfluidic device is then incubated in a water bath or incubator in preparation for the amplification and subsequent detection. (10) The color change is observed, and images are taken with a smartphone for further analysis.

### 2.9. Image Processing of the LAMP-μPAD Assay

A smartphone camera was utilized to capture amplification results from paper chips, followed by analysis using ImageJ software V1.54i. Images were decomposed into red (R), green (G), and blue (B) channels, with color intensity data extracted from designated detection zones. The colorimetric shift from yellow–orange to magenta was characterized by a significant increase in green intensity and a slight rise in red intensity. Consequently, green intensity served as the primary metric for quantifying amplification progression, with red intensity as a reference. The normalized G/R intensity was adopted as the standard measure throughout the study. All kinetic measurements were performed in quintuplicate (*n* = 5) under strictly controlled identical conditions to ensure reproducibility, with results presented as mean ± SD.

### 2.10. Determination of Diagnostic Cut-Off Values and Performance Evaluation of the LAMP-μPAD Assay

The threshold cut-off value was evaluated by receiver operator characteristic (ROC) analysis (MedCalc software V23.0.9) using 20 PEDV-positive and 20 negative-PEDV specimens, with RT-PCR as the reference method to classify all specimens.

To determine the optimal reaction kinetics of the LAMP-*μ*PAD assay, amplification efficiency was evaluated using PEDV cDNA as template. The time-dependent analysis was performed at 15-min intervals ranging from 15 to 40 min under isothermal conditions. The optimal reaction time was determined through analysis of colorimetric changes on the devices and 1% agarose gel electrophoresis results. The quantitative analysis of device images using ImageJ software established a correlation between reaction time and the normalized green-to-red intensity ratio (G/R). This relationship was graphically represented, elucidating the kinetics of the RT-LAMP reaction on the paper-based platform.

The optimized assay underwent rigorous specificity and sensitivity assessments to validate its potential as a reliable PEDV diagnostic tool. Specificity was evaluated using cDNA templates from diverse porcine pathogens, including PEDV, PDCoV, PCV2, PRRSV, and TGEV, with nuclease-free water as a negative control. Sensitivity was determined using 10-fold serial dilutions of PEDV cDNA (4.82 × 10^7^ to 4.82 × 10^1^ copies/μL). Both assessments employed a multi-faceted approach, combining visual colorimetric analysis, 1% agarose gel electrophoresis, and quantitative image analysis.

### 2.11. Clinical Implementation of the LAMP-μPAD Assay for PEDV Detection

To validate the diagnostic utility of the LAMP-*μ*PAD assay in clinical settings, we conducted a comparative analysis using 132 archived fecal specimens [24]. These specimens were simultaneously evaluated using both the developed LAMP-*μ*PAD and a reference RT-PCR method (see details in Supplementary Materials). Diagnostic accuracy parameters (sensitivity and specificity) were calculated with their corresponding exact binomial 95% confidence intervals (CIs). The diagnostic agreement between LAMP-*μ*PAD and RT-PCR was assessed using Cohen’s kappa coefficient [25].

### 2.12. Statistical Analysis

Statistical analyses were conducted using R version 4.3.3 (https://www.r-project.org/, accessed on 29 February 2024). The correlation between reaction time and G/R intensity were analyzed by using linear regression. Group comparisons were analyzed by a two-sided Student’ s *t*-test, and the difference was considered significant when *p* value *<* 0.05. Numbers of asterisks mean significant difference (* *p* < 0.05; ** *p* < 0.01; *** *p* < 0.001; **** *p* < 0.0001).

## 3. Results

### 3.1. Development of RT-LAMP Reaction System

**Figure 2:**
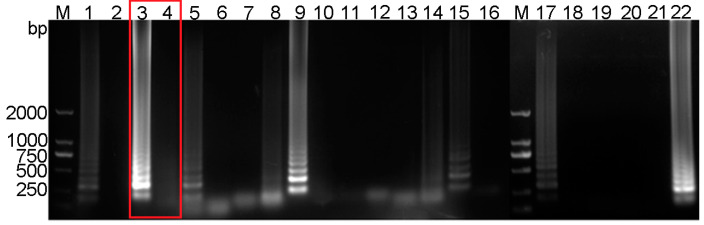
Screening of PEDV RT-LAMP primer sets. M for 2000 bp DNA marker, lanes 1 and 2 represent the positive and negative amplification results of primer set 1, respectively; lanes 3 and 4 for primer set 2; lanes 5 and 6 for primer set 3; lanes 7 and 8 for primer set 4; lanes 9 and 10 for primer set 5; lanes 11 and 12 for primer set 6; lanes 13 and 14 for primer set 7; lanes 15 and 16 for primer set 8; lanes 17 and 18 for primer set 9; lanes 19 and 20 for primer set 10; lanes 21 and 22 for primer set 11. Red rectangular frames highlight primer set 2, which was selected as the optimal primer pair based on specificity and amplification efficiency analyses.

**Figure 3:**
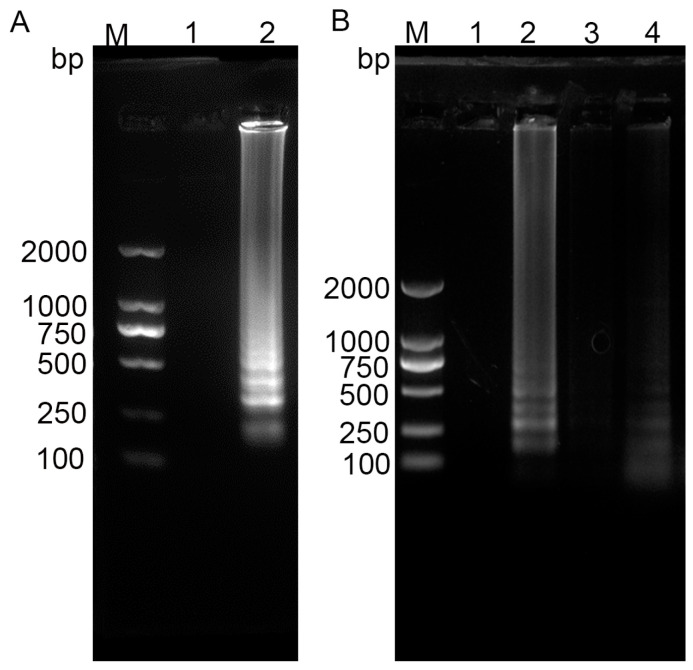
Development of RT-LAMP assay and application on different types of paper. (**A**) RT-LAMP for PEDV testing. M: DL2000 DNA marker; 1: negative control (ddH_2_O); 2: PEDV positive. (**B**) Comparative analysis of paper types for nucleic acid amplification zone. M: DL2000 DNA marker; 1 and 2 represent PEDV negative and positive amplification using RT-LAMP on Whatman No. 1 filter paper, respectively; 3 and 4 represent PEDV negative and positive amplification using RT-LAMP on NC membrane, respectively. (*n* = 5).

Electrophoresis analysis of 11 RT-LAMP primer sets for PEDV detection was performed, revealing that primer set #2 (boxed in red, Figure 2) exhibited optimal performance. This primer set was selected for downstream experiments based on its strong target-specific amplification (lanes 3–4). The primer sequences of primer set #2 were as follows: F3 (forward outer primer): 5′-TCAACAAGTTTCACAAAAACC-3′, B3 (backward outer primer): 5′-ATGGCTTCAAGCAATGCA-3′; FIP (forward inner primer): 5′-CTTCAGGGTTGCACTCATAGAAATTTGCCTAATTTTGAACCTTTCA-3′, BIP (backward inner primer): 5′-TAGGTGCTGACAAGCTGGTGGGTCACATTGTTAAGACACTT-3′ (Figure S1)**.** The optimized reaction time and temperature were determined to be 30 min and 60 °C, respectively. The inner primer concentration was found to be optimal at 32 μM, and the outer primer concentration was determined to be 5 μM for subsequent experiments. The Bst enzyme concentration was optimized at 10,000 U/mL, at which point the band intensity reached its maximum. Furthermore, the optimal concentrations for Mg^2+^, dNTPs, and buffer were established at 80 mM, 10 mM, and 0.8-times dilution, respectively (Figure S1). Based on these findings, a final RT-LAMP reaction system was developed (Figure 3A).

### 3.2. Selection of Paper Substrates

Comparative analyses of LAMP were conducted on two distinct substrates: Whatman No. 1 filter paper and NC membranes. The results demonstrated superior amplification efficiency on Whatman No. 1 filter paper relative to NC membranes, as evidenced by enhanced signal intensity and reaction uniformity (Figure 3B). Based on these findings, Whatman No. 1 filter paper was selected as the optimal substrate for the nucleic acid amplification zone, owing to its balanced hydrophilicity, porosity, and compatibility with LAMP reagent immobilization.

### 3.3. Screening of Nucleic Acid Lysis Buffer

**Figure 4:**
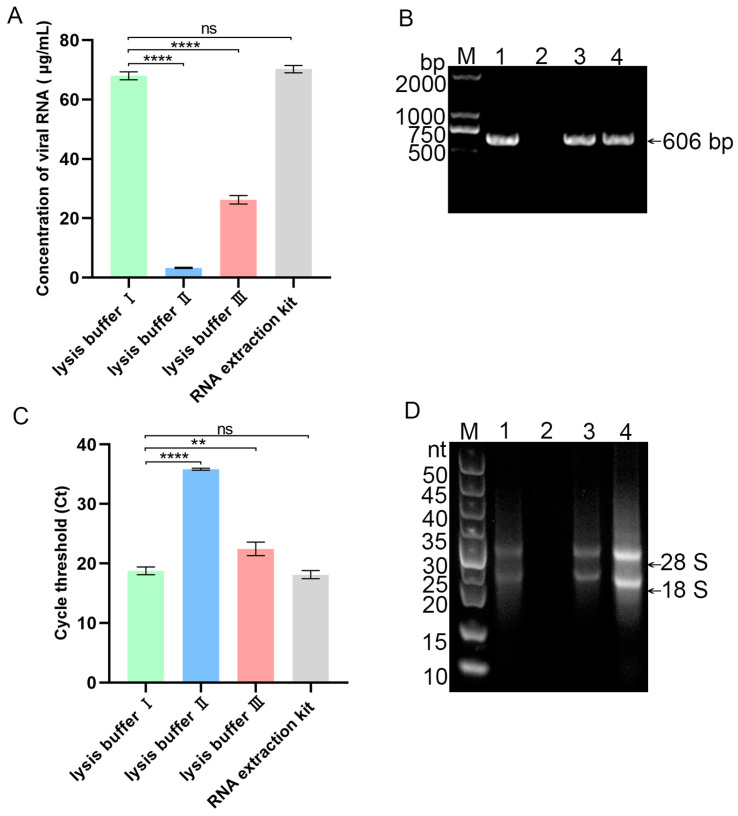
Screening of nucleic acid lysis buffers. (**A**) Concentration measurements using ultra-micro nucleic acid protein analyzer. (**B**) RT-PCR results. M: DL2000 DNA marker; Lane 1: Lysis buffer I; Lane 2: Lysis buffer II; Lane 3: Lysis buffer III; Lane 4: RNA extraction kit. (**C**) qPCR results. (**D**) RNA agarose gel electrophoresis results. Lane 1: Lysis buffer I; Lane 2: Lysis buffer II; Lane 3: Lysis buffer III; Lane 4: RNA extraction kit. (* indicates comparison between groups, ns denotes non-significant differences, ** p < 0.01, **** p < 0.0001, *n* = 3).

The PEDV solution was divided into three aliquots, each treated with lysis buffer I, II, and III for viral nucleic acid lysis, respectively, followed by enrichment using a glass fiber membrane. The extracted nucleic acids underwent comprehensive analysis via RT-PCR, qPCR, and RNA agarose gel electrophoresis. The results revealed that lysis buffer I produced the highest viral RNA concentration among the three buffers tested, as demonstrated by the most pronounced target bands observed after RT-PCR amplification (Figure 4A,B). Comparative qPCR analysis revealed Ct values of approximately 19, 35, 23, and 18 for viral nucleic acids extracted using lysis buffers I, II, III, and commercial RNA extraction kit, respectively (Figure 4C). RNA gel electrophoresis demonstrated that lysis buffers I and III achieved RNA integrity comparable to the commercial kit, as indicated by distinct 28S and 18S bands (Figure 4D). Despite this, buffer I was selected due to its superior nucleic acid recovery (lower Ct values: ~19 for buffers I vs. ~23 for buffers III), enhanced compatibility with LAMP through optimized detergent/proteinase K composition, and greater reproducibility (smaller inter-replicate variability). Furthermore, lysis buffer I’s cost-effectiveness (USD 0.12/sample vs. USD 2.50–5.00 for commercial kits), non-proprietary formulation, and alignment with WHO ASSURED criteria make it particularly suitable for point-of-care applications in resource-limited settings.

### 3.4. Optimization of the Channel and Nucleic Acid Amplification Zone Geometry

**Figure 5:**
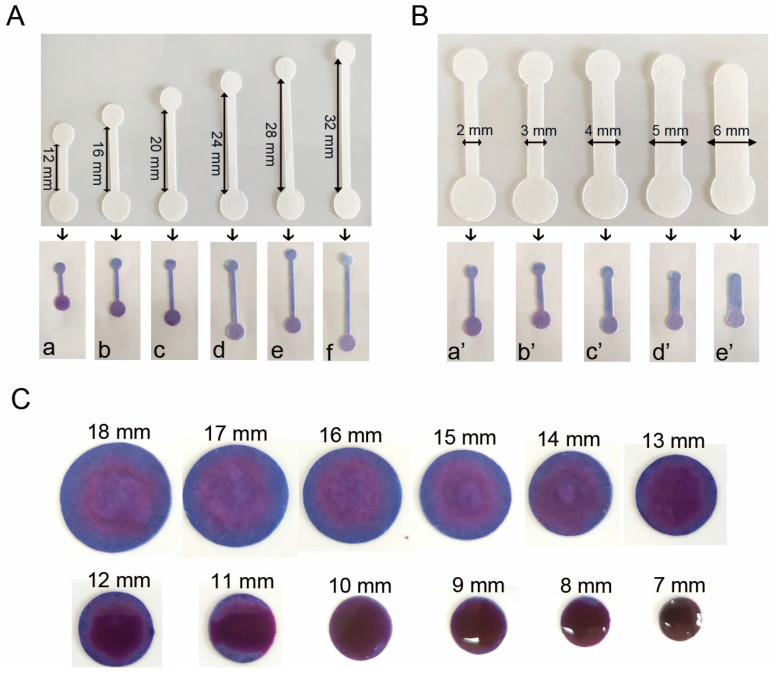
Optimization of nucleic acid chromatography channel geometry. (**A**) Channel length optimization (**a**–**f**: 12, 16, 20, 24, 28, and 32 mm, respectively). (**B**) Channel width optimization (**a’**–**e’**: 2, 3, 4, 5, and 6 mm, respectively). (**C**) Dimensional optimization of amplification reaction chambers for nucleic acid detection. (*n* = 5).

For channel length, tests on 12 mm to 32 mm lengths revealed that 16 mm achieved complete fluid penetration in 10 s with minimal residual ink, making it optimal (Figure 5A). Channel width optimization (2 mm to 6 mm) identified 4 mm as ideal, enabling full penetration in 12 s with the least residual ink (Figure 5B). For the dimensions of nucleic acid amplification zone, the results indicated that a chamber diameter of 13 mm provided optimal performance. At this dimension, the blue ink precisely filled the chamber with minimal residual volume (Figure 5C). Consequently, a reaction chamber with a diameter of 13 mm was selected as the optimal configuration for the nucleic acid amplification zone in the paper-based chip design. These optimizations enhanced fluid flow efficiency, reduced mixing, and improved detection sensitivity and accuracy.

### 3.5. Selection of Colorimetric Indicators for the LAMP-μPAD Assay

**Figure 6:**
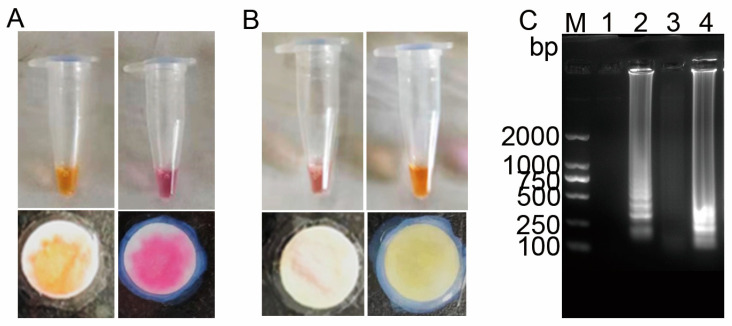
Comparison of colorimetric indicators. (**A**) Neutral red and (**B**) cresol red color transitions before and after LAMP amplification. (**C**) Agarose gel electrophoresis analysis. Lane M: DL2000 DNA marker; Lanes 1 and 2: electrophoresis results of neutral red before and after LAMP amplification, respectively; Lanes 3 and 4: electrophoresis results of cresol red before and after LAMP amplification, respectively (*n* = 5).

To enhance the colorimetric detection system, the standard isothermal amplification buffer was replaced with a weak buffer (pH 8.0). Cresol red and neutral red were evaluated as potential indicators for both conventional and paper-based RT-LAMP assays. Assessment criteria included colorimetric transitions and amplicon detection via agarose gel electrophoresis. Both dyes exhibited distinct color changes in solution-based RT-LAMP (Figure 6A,B), with gel electrophoresis confirming specific amplification (Figure 6C). In paper-based assays, while both dyes demonstrated color shifts, cresol red’s water solubility led to substrate diffusion, compromising quantitative analysis (Figure 5B). Conversely, neutral red’s oil solubility minimized diffusion while maintaining clear color transitions (Figure 5A). Consequently, neutral red was selected as the optimal indicator for paper-based RT-LAMP, offering superior stability and visual clarity.

### 3.6. Cut-Off Value and Diagnostic Performance Evaluation of the LAMP-μPAD Assay

**Figure 7:**
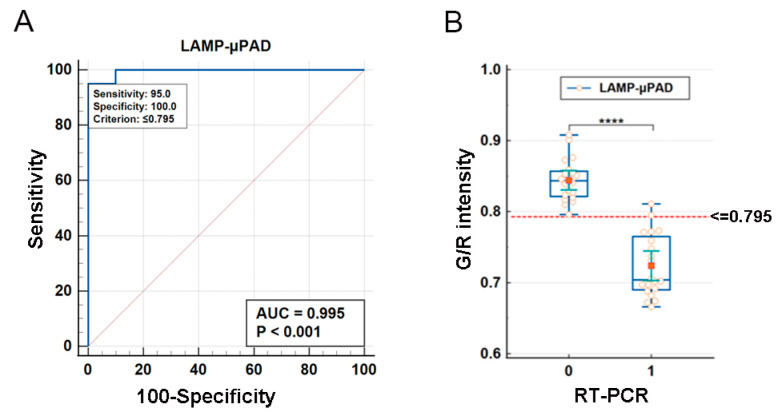
Cut-off value and diagnostic performance evaluation of the LAMP-*μ*PAD assay. (**A**) Determination of Cut-off value. (**B**) ROC curve for diagnostic performance evaluation. (**** p < 0.0001).

A total of 20 clinically validated PEDV-positive and 20 PEDV-negative fecal specimens were subjected to both LAMP-*μ*PAD and RT-PCR assays. The results demonstrated a significant difference in the G/R intensity between PEDV-positive and negative specimens (*p* < 0.001). Based on the ROC curve analysis of the LAMP-*μ*PAD, the G/R intensity of the 20 PEDV-positive specimens varied from a minimum of 0.666 to a maximum of 0.811, and from 0.796 to 0.908 for the 20 PEDV-negative specimens. The G/R intensity of 0.795 was selected as the optimal cut-off value, with an area under the curve (AUC) of 0.995 ± 0.00607 and 95% confidence interval (CI) ranging from 0.902 to 1.000 (Figure 7).

### 3.7. Reaction Time for the LAMP-μPAD Assay

**Figure 8:**
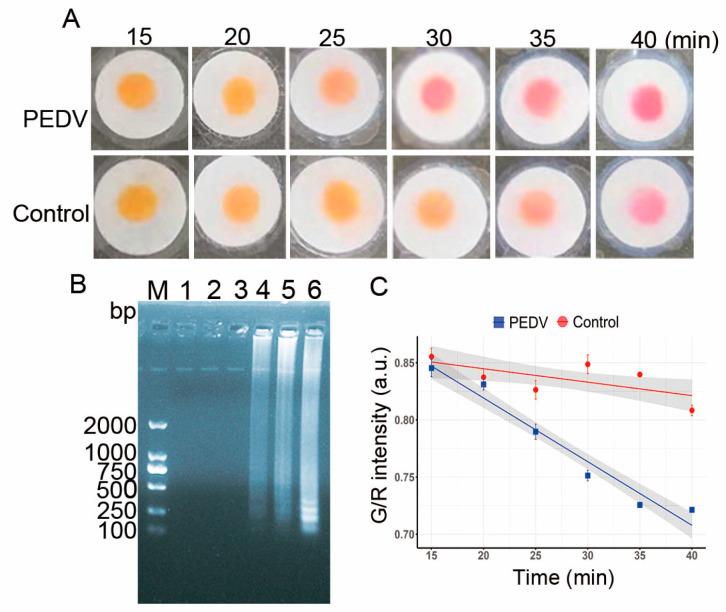
Time-dependent analysis of the LAMP-*μ*PAD assay. (**A**) Colorimetric results on paper chips for PEDV and negative control. (**B**) Time course analysis by agarose gel electrophoresis. M: DL2000 DNA marker; Lanes 1–6 represent 15, 20, 25, 30, 35, and 40 min, respectively. (**C**) Regression analysis of G/R intensity versus reaction time (error bars represent standard deviation from five replicates for each time point) (*n* = 5).

Following the previously optimized conditions, we sought to determine the optimal reaction time for the LAMP-*μ*PAD assay. As illustrated in Figure 8A, a distinct color transition from yellow–orange to magenta was observed in the positive specimens at the 30-min mark, while the negative controls remained unchanged. Corroborating these visual results, gel electrophoresis analysis revealed clearly discernible bands at 30 min (Figure 8B). Furthermore, a colorimetric response intensity analysis using ImageJ software demonstrated a significant disparity in the G/R intensity between positive and negative specimens at 30 min. Notably, the G/R intensity of positive specimens exhibited a linear correlation with reaction time (Figure 8C). These findings indicate that amplification commenced at approximately 25 min, with a pronounced color change manifesting at 30 min. Consequently, the optimal reaction time for the paper-based assay was determined to be 30 min.

### 3.8. Specificity and Sensitivity of the LAMP-μPAD Assay

**Figure 9:**
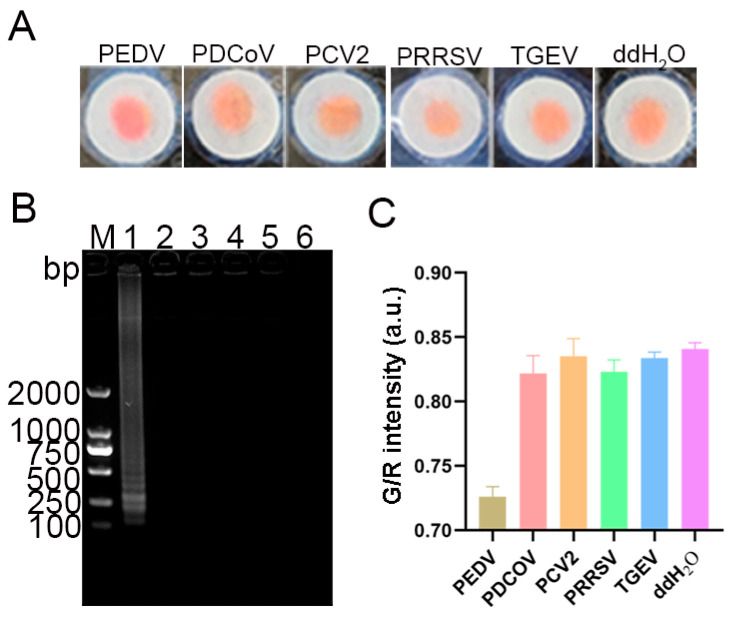
Specificity analysis of the LAMP-*μ*PAD assay. (**A**) Visual colorimetric detection results on paper chips. (**B**) Agarose gel electrophoresis analysis. M: DL2000 DNA marker; Lanes 1–6 represents PEDV, PDCoV, PCV2, PRRSV, TGEV and ddH_2_O, respectively. (**C**) Quantitative analysis of colorimetric intensity (G/R).

As illustrated in Figure 9A, a distinct color transition from yellow–orange to magenta was observed exclusively for the PEDV-positive specimen. The paper chips for other viruses and the negative control maintained their original yellow–orange hue. Corroborating these findings, the agarose gel electrophoresis results revealed characteristic ladder-like bands only in the PEDV lane, while other lanes remained devoid of amplification products (Figure 9B). Further quantitative analysis of the G/R intensity using ImageJ software demonstrated that the PEDV-positive specimen had a significantly lower G/R intensity compared to other viruses and the negative control (Figure 9C). This marked difference in colorimetric intensity further substantiates the specificity of the assay. Collectively, these results provide compelling evidence for the high specificity of the developed LAMP-*μ*PAD method for PEDV detection.

**Figure 10:**
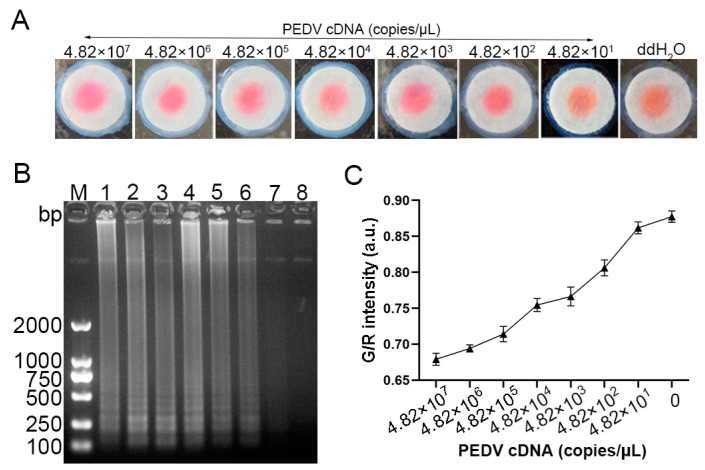
Sensitivity evaluation of the LAMP-*μ*PAD assay. (**A**) Visual detection results on the paper chips at different template concentrations. (**B**) Agarose gel electrophoresis analysis. M: DL2000 DNA marker; Lanes 1–7: Serial dilutions of template from 4.82 × 10^7^ to 4.82 × 10^1^ copies/μL; Lane 8: negative control (ddH_2_O). (**C**) G/R ratio changes across plasmid concentration gradients. (*n* = 5).

The sensitivity of the LAMP-*μ*PAD assay was evaluated through colorimetric analysis and gel electrophoresis. As illustrated in Figure 10A, a distinct chromatic shift from yellow–orange to magenta was observed at PEDV cDNA concentration threshold of 4.82 × 10^2^ copies/μL. This color transition ceased at 4.82 × 10^1^ copies/μL, a finding that was corroborated by agarose gel electrophoresis results (Figure 10B). These findings indicate that the limit of detection for the paper-based assay was 4.82 × 10^2^ copies/μL. Further quantitative analysis using ImageJ software unveiled an inverse relationship between the G/R intensity and template concentration. A progressive decrease in the G/R intensity was observed with increasing cDNA concentrations, demonstrating a linear correlation (Figure 10C).

### 3.9. Application of the LAMP-μPAD in Clinical Validation

A total of 132 clinical specimens were analyzed using both RT-PCR and the LAMP-*μ*PAD assay. RT-PCR analysis identified 43 PEDV-positive and 89 PEDV-negative specimens, corresponding to a positivity rate of 32.6% (43/132). The LAMP-*μ*PAD detected 41 PEDV-positive and 91 PEDV-negative specimens, yielding a positivity rate of 31.1% (41/132). Among these results, 41 specimens were concordantly positive by both methods. However, two specimens that positive tested by RT-PCR yielded negative results with the LAMP-*μ*PAD. All 89 specimens that tested negative by RT-PCR were also negative by LAMP-*μ*PAD. Compared with RT-PCR, the specificity and sensitivity of the LAMP-*μ*PAD were 100% (95% CI: 91.8–100%) and 95.3% (95% CI: 86.2–98.8%), respectively, with a concordance rate of 98.5% and a kappa value of 0.965 (Table 1). These findings indicate that the LAMP-*μ*PAD assay developed in this study possesses good clinical detection capabilities and can be employed for PEDV clinical testing.

## 4. Discussion

PEDV has emerged as a significant viral pathogen causing severe diarrheal disease in neonatal piglets. The global dissemination of PEDV in swine populations has inflicted considerable economic losses, fundamentally disrupting the swine industry’s development [26,27]. Although vaccination strategies have been implemented, recurring PED outbreaks underscore the urgent demand for rapid, sensitive diagnostic platforms. Recent advances in isothermal amplification techniques, lateral flow assays, and microfluidic platforms have revolutionized PEDV detection strategies [28,29]. These innovative diagnostic approaches, characterized by rapid turnaround times, high analytical sensitivity, exceptional specificity, and visual readout capabilities, establish a compelling framework for advancing clinical PEDV diagnostics.

Paper-based microfluidics offers advantages over traditional microfluidic systems by addressing challenges like sample processing and fluid control. Paper serves as an ideal material for transitioning from lab to consumer use, enabling liquid transport through capillary action without pumps. This technology aligns with WHO’s ASSURED criteria for point-of-care diagnostics by being affordable, sensitive, specific, user-friendly, rapid, equipment-free, and deliverable to end-users [30]. The selection of paper substrates for *μ*PAD critically influences their performance in terms of fluid flow dynamics, reagent compatibility, and detection sensitivity. Optimal paper choice requires balancing key parameters such as porosity, thickness, and material composition—for instance, cellulose for cost-effectiveness or nitrocellulose for enhanced protein-binding capacity. Compatibility with chemical reagents and fabrication techniques (e.g., wax patterning, precision cutting, or laser ablation) must be carefully evaluated. The inherent hydrophilicity of cellulose-based materials facilitates capillary-driven flow, while surface functionalization through hydroxyl groups enables covalent reagent immobilization. Cost-effective options like Whatman No. 1 filter paper are preferred for general applications, whereas nitrocellulose membranes provide high analytical sensitivity, and glass fiber substrates enable rapid fluid transport [31,32]. In this study, we engineered a 3D foldable paper-based microfluidic chip that leverages glass fiber to concentrate viral nucleic acids. We utilized Whatman No. 1 filter paper as both the chromatographic channel and the substrate for the LAMP amplification reaction, achieving excellent detection performance for PEDV. The extraction and enrichment of viral nucleic acid are pivotal steps in the design of *μ*PAD that enhance the efficiency, sensitivity, and reliability of pathogen detection in various applications, particularly in low-resource or acute settings where rapid results are essential [33,34]. To enable streamlined sample pretreatment in point-of-care testing (POCT) applications, we developed a novel paper-based lysis and enrichment method that facilitates rapid nucleic acid extraction directly from fecal specimens. Through systematic optimization of nucleic acid lysis buffer formulations, we enhanced both RNA yield and extraction stability. The optimized lysis buffer I (4.5 M guanidine thiocyanate, 50 mM Tris-HCl, 30% Triton X-100, and 1 mg/mL proteinase K) demonstrated comparable performance to commercial RNA extraction kits in processing viral targets while maintaining lower cost and non-toxic composition—a critical advantage for decentralized POC diagnostic implementations. This integrated pretreatment method demonstrated exceptional sensitivity (4.82 × 10^2^ copies/μL) in downstream LAMP-*μ*PAD assays, matching the detection limits of conventional column-based purification systems.

The geometric configuration of *μ*PAD chromatographic channels critically determines detection performance. Narrower channels impede fluid flow due to inadequate capillary forces, whereas wider channels enhance flow dynamics at the expense of analyte mixing [35]. Channel length directly modulates detection time, with extended lengths delaying signal acquisition [28]. Advanced fabrication techniques (e.g., precision cutting, hot-pressing) enable optimized detection zone architectures that improve sensitivity and reproducibility. Mitigating evaporation through reduced exposed surface area and enhancing structural integrity collectively ensure operational reliability [36]. In the present study, we systematically optimized the channel length, width, and reaction chamber size of paper-based microfluidic chips to improve detection performance. These optimizations enhanced fluid flow efficiency, reduced mixing, and improved detection sensitivity and accuracy. When compared with a recently reported 3D-printed RT-LAMP microfluidic chip for multiplex detection of PEDV, TGEV and PDCoV [29], our LAMP-*μ*PAD platform shows lower sensitivity (4.82 × 10^2^ copies/μL vs. 10^1^ copies/reaction for PEDV), but it offers significant practical advantages over photopolymer resin-based 3D-printed chips, particularly in terms of simplified fabrication processes and substantially lower production costs.

One limitation of our study is that we did not extensively investigate the long-term stability and durability of the LAMP-*μ*PAD assay under varying environmental conditions and various biological samples (tap water, feed, urine, and blood plasma samples, etc.), which could affect its practical utility. Future research should address this limitation to further enhance the robustness and applicability of this diagnostic tool. Nevertheless, to the best knowledge, this investigation represents the first reported application in the literature of a paper-based sensor for PEDV test. It provides an innovative technological framework for rapid and effective on-site diagnosis, thereby enhancing timely disease management and control. Notably, distinct banding patterns were observed across different substrates (Whatman No. 1, NC membrane, and standard RT-LAMP system), suggesting substrate-dependent effects on amplification efficiency. These variations likely stem from a complex interplay of: (1) substrate-specific nucleic acid retention characteristics that modulate template availability and local concentration gradients during amplification; (2) differential non-covalent interactions between paper matrices and DNA fragments (particularly during elution) that may sterically hinder polymerase access or promote alternative priming; (3) competitive binding of reaction components to cellulose fibers that alter effective primer/template ratios; and (4) substrate-dependent formation of primer-dimers or other amplification byproducts due to microenvironmental effects on reaction kinetics, as evidenced by the characteristic ~100 bp band appearing only in specific substrate conditions. These phenomena collectively demonstrate how physical substrate properties can fundamentally influence LAMP reaction fidelity and efficiency through both direct chemical interactions and spatial organization of reaction components. In the future, systematic optimization of substrate material properties (e.g., pore size, surface charge, and chemical modifications) should be conducted to mitigate these matrix effects while maintaining field-deployable simplicity. Additionally, real-time monitoring of amplification kinetics on different substrates could help elucidate the precise mechanisms underlying these substrate-dependent performance variations, enabling rational design of next-generation μPAD platforms.

## 5. Conclusions

In summary, this study establishes a novel paper-based LAMP platform for rapid and precise detection of PEDV. The platform synergistically integrates nucleic acid enrichment, amplification, and visual lateral flow detection, identifying PEDV in under 30 min. This cost-effective and user-friendly *μ*PAD addresses the urgent need for PEDV on-site diagnostics in resource-limited settings, offering a practical alternative to traditional methods. The design’s flexibility also hints at detecting other pathogens, expanding its value in veterinary and public health diagnostics.

## Figures and Tables

**Table 1:** Comparison between the LAMP-*μ*PAD and RT-PCR for detecting PEDV.

		RT-PCR		Total
		Positive	Negative	
LAMP-*μ*PAD	Positive	41	0	41
	Negative	2	89	91
	Total	43	89	132

## Data Availability

Data are contained within the article and Supplementary Materials.

## Supplementary Materials

The following supporting information can be downloaded at: https://www.mdpi.com/article/10.3390/vetsci12050427/s1, Figure S1: optimization of the PEDV RT-LAMP reaction system. (**A**) Reaction time (lanes 1–8 correspond to 10, 20, 30, 40, 50, 60, 70, and 80 min, respectively); (**B**) reaction temperature (lanes 1–8 correspond to 54, 56, 58, 60, 62, 64, 66, and 68 °C, respectively); (**C**) Mg^2+^ concentration (lanes 1–8 correspond to 0, 20, 40, 60, 80, 100, 120, and 140 mM, respectively); (**D**) inner primer concentration (lanes 1–8 correspond to 8, 16, 24, 32, 40, 48, 56, and 64 μM, respectively); (**E**) outer primer concentration (lanes 1–8 correspond to 1, 2, 3, 4, 5, 6, 7, and 8 μM, respectively); (**F**) dNTPs concentration (lanes 1–8 correspond to 4, 5.5, 7, 8.5, 10, 11.5, 13, and 14.5 mM, respectively); (**G**) Bst enzyme concentration (lanes 1–8 correspond to 1000, 2000, 4000, 6000, 8000, 10,000, 12,000, and 14,000 U/mL, respectively); (**H**) buffer dilution (lanes 1–8 correspond to 0.4, 0.6, 0.8, 1.0, 1.2, 1.4, 1.6, and 1.8-times dilution, respectively); Table S1: the information of the primers used for RT-LAMP.
