# Supplementary material for: Development of a Paper-Based Microfluidic Chip for Point-of-Care Detection of PEDV
Source: Vet Sci. 2025 Apr 30;12(5):427. doi: 10.3390/vetsci12050427 (PMC12115601; doi:10.3390/vetsci12050427)
Supplement: Supplementary file 1 [file vetsci-12-00427-s001.zip › vetsci-3562432-supplementary.pdf]

veterinary sciences

Supplementary Materials

Development of a Paper-Based Microfluidic Chip for Point-of-Care Detection of PEDV

Renfeng Li [1], Xiangqin Tian [2], Wenyan Cao [1], Jiaxin Jiang [1], Jiakang Yuan [1], Linyue Li [1], Yonghe You [3], Yanlin Zhou [3], Ziliang Wang [1,*] and Fangyu Wang [4,*]

1 College of Animal Science and Veterinary Medicine, Henan Institute of Science and Technology, Xinxiang 453003, China; lirenfeng@sina.com (R.L.); caowenyan@stu.hist.edu.cn (W.C.); jiangjiaxin@stu.hist.edu.cnJ.J.); yjkhist2024@163.com (J.Y.); m18236356245@163.com (L.L.)
2 Henan Key Laboratory of Medical Tissue Regeneration, Xinxiang Medical University, Xinxiang 453003, China; tianxiangqin@xxmu.edu.cn
3 Sanquan College, Xinxiang Medical University, Xinxiang 453003, China; sqjcyyh2013@126.com (Y.Y.); zhouyanlin@xxmu.edu.cn (Y.Z.)
4 Key Laboratory of Animal Immunology, Institute for Animal Health, Henan Academy of Agricultural Sciences, Zhengzhou 450002, China
* Correspondence: wziliang2000@163.com (Z.W.); sprinkle.w@126.com (F.W.)

Primer Design

Based on the ORF1a/b gene sequence of the PEDV strain CH/HNQX-3/14 isolated in our laboratory (GenBank accession number: KR095279.1), 11 primer sets were designed for LAMP amplification using Primer Premier 5.0 software. Each primer set comprises an outer pair (F3/B3) and an inner pair (FIP/BIP). All primers were synthesized by Shanghai Sangon Biotechnology Co., Ltd. The sequences of each primer set are presented in Table S1.

Table S1. The information of the primers used for RT-LAMP.

Primer sets[a]		Primer sequences (5′→3′)
PE1	F3	TGATACTGAGTGTGACAAGTT
	B3	CACAGATTGTAGGTGTCAAAC
	FIP	AGCTCCACCAACATTACATTTAGTACAGGACTCTATAAACTACGTTCC
	BIP	TGTAGTAAGCATTGTGCTATGTACCCCCAAATCGTAAAGCCCG
PE2	F3	TCAACAAGTTTCACAAAAACC
	B3	ATGGCTTCAAGCAATGCA
	FIP	CTTCAGGGTTGCACTCATAGAAATTTGCCTAATTTTGAACCTTTCA
	BIP	TAGGTGCTGACAAGCTGGTGGGTCACATTGTTAAGACACTT
PE3	F3	ACGTTGTATTCCAAGTTGTC
	B3	ACGGTGTCATTAACAAGACA
	FIP	ATTTGCGTAGAAACGCTCCACCCATCTCAGTGTGGTAGGT
	BIP	AGTCACTGAGGATACACGTAGTGGGTCCAATTTGTTGTCCAT
PE4	F3	ACCTGTACAATCCATACTGTA
	B3	TTAATAACAGCCTCATTACCAAT
	FIP	GCCACATGCTCGTTTCTATGTACCAGTGGGGATACAAGGGAT
	BIP	GATGCCATAATGACTCGCTGTTGGGTATGTGATGGACCAG
PE5	F3	CGCACTGTGTCTGAGATG
	B3	ATTCTGGCTGTTATAAGGAGA
	FIP	CTGAACATTACCCTTGCAAAAGATTGTCTACGAAAACCAATTCATTCC
	BIP	TCAAGCATTAATCGCAGGCAAAATAAAAACAGCCTTTGACCA
PE6	F3	GTTGTTTGTTACAACAATGACT
	B3	GATTCTTGAAGGATCAGGGTA
	FIP	GCTCATGAAGACATTGTTCTGGTAACATCACTTGGTTATGTCGCT
	BIP	GCCTGACATTAATAAAGGTCCTCATAGGTAGGTAGTAAGTACCATC
PE7	F3	TTCTGGCGTAATTCCACA
	B3	ACCAAATGATTAGAAAAACCACA

	FIP	ACTGTAGCCTTATGCTTACAATTGAATGACTATGTCTTACAATAGTGTA
	BIP	ATCCATTAGTGATGTTGTGTTAGGTCGTCATTATTACGCACTAGC
PE8	F3	CCCAGTTAACACACATGAAGA
	B3	CGTGCTCAAAGCCGTAAT
	FIP	AGCCATCAGGATAGTCCTCGACCTTTTACTTGGTACATCTACAC
	BIP	CCGCTGATTTTAGCCCTCGTAGTCCGTACTTGTTAATAAACAGA
PE9	F3	CTAATGAGGGTTGTGGTCT
	B3	TTGATATCAAAACGGAAGCC
	FIP	GACATGAAGGTGTTAGCGTGAGTTTAAAGACTGTAGCAGAGGTG
	BIP	TAGCGGACAATTTTAAGACTGATCAACATGCTCATATTTAATGGGTC
PE10	F3	GTCAGCCTAGTGATTCAGA
	B3	TTGACAACAATTGTAGTACCTT
	FIP	CGCTTACTACAACAACAGCCGTGTCCAAGTACATTGTTTCTGC
	BIP	ACTGCACCAGTTGTGAATGCTATGTCAATGTAGTTAAGACCATG
PE11	F3	TGGTCGTGCTATCTATGGTA
	B3	TGCATAGACACGATGAATGT
	FIP	CGCAATTGTTTTCATCAAAGTTACGACGTTTGTAGAAAGGATCTTACC
	BIP	AGCATTTTAATTAAGGTAGGCGCTCTTCATTTTCAACAGGGTCAA

[a]FIP / BIP: inner primer set; F3 / B3: outer primer set.

Optimization of RT-LAMP System

We undertook an optimization process for various parameters implicated in the LAMP reaction, including reaction time (10 min, 20 min, 30 min, 40 min, 50 min, 60 min, 70 min, and 80 min), reaction temperature (54 ℃, 56 ℃, 58 ℃, 60 ℃, 62 ℃, 64 ℃, 66 ℃, and 68 ℃), concentrations of inner primers (8 μM, 16 μM, 24 μM, 32 μM, 40 μM, 48 μM, 56 μM, and 64 μM) and outer primers (1 μM, 2 μM, 3 μM, 4 μM, 5 μM, 6 μM, 7 μM, and 8 μM), Bst enzyme concentration (1, 000 U/mL, 2, 000 U/mL, 4, 000 U/mL, 6, 000 U/mL, 8, 000 U/mL, 10, 000 U/mL, 12, 000 U/mL, and 14,000 U/mL), Mg^{2+} concentration (0 mM, 20 mM, 40 mM, 60 mM, 80 mM, 100 mM, 120 mM, and 140 mM), dNTPs concentration (1-8 represent 4 mM, 5.5 mM, 7 mM, 8.5 mM, 10 mM, 11.5 mM, 13 mM, and 14.5 mM),, and buffer concentration (dilution of 0.4 ×, 0.6 ×, 0.8 ×, 1.0 ×, 1.2 ×, 1.4 ×, 1.6 ×, and 1.8 ×). The optimal conditions were determined based on agarose gel electrophoresis results. The final optimized conditions are as follows: reaction 50 min (Figure S1A) at 60 ℃ (Figure S1B), the concentration of Mg^{2+} was 80 mM (Figure S1C), the concentrations of inner primer and outer primer were 32 μM (Figure S1D) and 5 μM (Figure S1E), respectively. The optimal concentrations of Bst enzyme, dNTPs and buffer were found to be 10,000 U/mL (Figure S1F), 10 mM (Figure S1G), and 0.8× (Figure S1H), respectively.

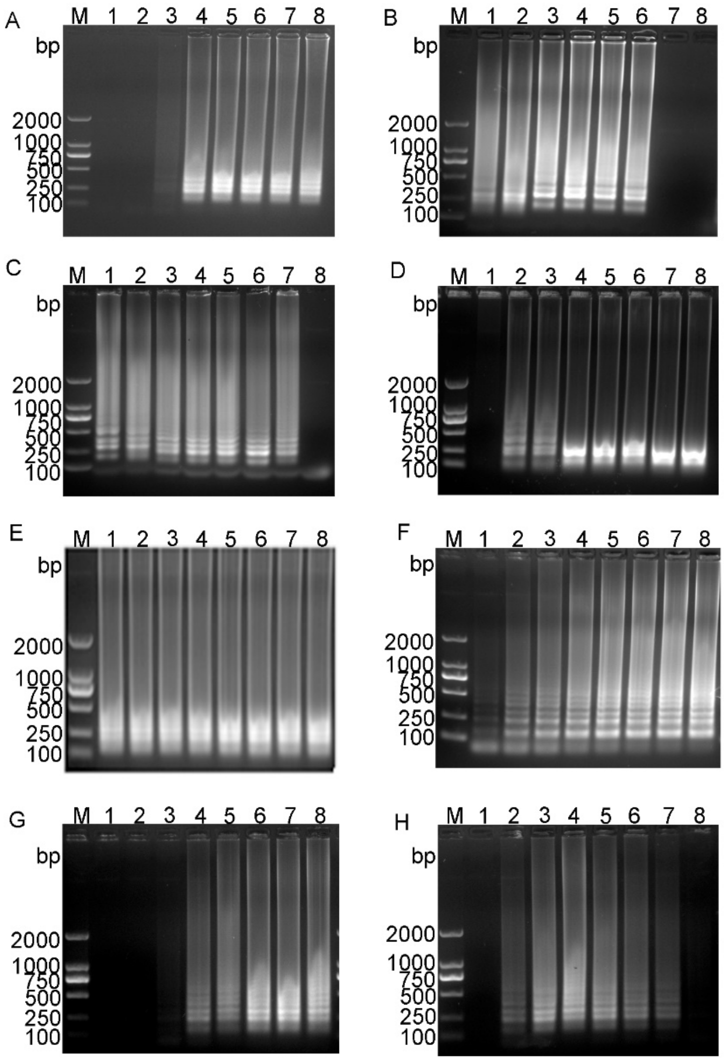

Figure S1. Optimization of the PEDV RT-LAMP reaction system. **(A)** Reaction time (lanes 1-8 correspond to 10, 20, 30, 40, 50, 60, 70, and 80 min, respectively); **(B)** Reaction temperature (lanes 1-8 correspond to 54, 56, 58, 60, 62, 64, 66, and 68 °C, respectively); **(C)** Mg^{2+} concentration (lanes 1-8 correspond to 0, 20, 40, 60, 80, 100, 120, and 140 mM, respectively); **(D)** Inner primer concentration (lanes 1-8 correspond to 8, 16, 24, 32, 40, 48, 56, and 64 μM, respectively); **(E)** Outer primer concentration (lanes 1-8 correspond to 1, 2, 3, 4, 5, 6, 7, and 8 μM, respectively); **(F)** dNTPs concentration (lanes 1-8 correspond to 4, 5.5, 7, 8.5, 10, 11.5, 13, and 14.5 mM, respectively); **(G)** Bst enzyme concentration (lanes 1-8 correspond to 1000, 2000, 4000, 6000, 8000, 10000, 12000, and 14000 U/mL, respectively); **(H)** Buffer dilution (lanes 1-8 correspond to 0.4, 0.6, 0.8, 1.0, 1.2, 1.4, 1.6, and 1.8-times dilution, respectively).

RT-PCR

RT-PCR analysis was performed following the National Standard protocol (GB/T 34757-2017) established by the Ministry of Agriculture and Rural Affairs of the People's Republic of China. Briefly, fecal specimens were suspended in a five-fold volume of sterile phosphate-buffered saline (PBS) and centrifuged to remove debris. The supernatant was subsequently filtered through a 0.45 μm membrane filter. Viral RNA was isolated from the filtered supernatant using a Viral RNA Mini Kit (QIAGEN, Hilden, Germany) according to the manufacturer's instructions. First-strand cDNA synthesis was performed using

the PrimeScriptII 1st-strand cDNA synthesis kit (TaKaRa Bio, Kusatsu, Japan). The amplification was conducted using primers targeting the PEDV M gene sequence: forward primer (5'-TATGGCTTGCATCACTCTTA-3') and reverse primer (5'-TTGACTGAACGACCAACACG-3'). The thermal cycling conditions comprised initial denaturation at 95 °C for 5 min, followed by 30 cycles of denaturation at 94 °C for 30 s, annealing at 52.5 °C for 30 s, and extension at 72 °C for 30 s, with a final extension step at 72 °C for 10 min.
